# Liver injury in children: signal analysis of suspected drugs based on the food and drug administration adverse event reporting system

**DOI:** 10.1186/s12887-023-04097-9

**Published:** 2023-09-28

**Authors:** Yan Liu, Hailong Li, Liang Huang, Chaomin Wan, Huiqing Wang, Xuefeng Jiao, Linan Zeng, Zhijun Jia, Guo Cheng, Lei Zhang, Wei Zhang, Lingli Zhang

**Affiliations:** 1 Department of Pharmacy, West China Second University Hospital, Sichuan University, Chengdu, 610041 China; 2 Evidence-Based Pharmacy Center, West China Second University Hospital, Sichuan University, Chengdu, 610041 China; 3 NMPA Key Laboratory for Technical Research on Drug Products In Vitro and In Vivo Correlation, Chengdu, 610041 China; 4 Key Laboratory of Birth Defects and Related Diseases of Women and Children, Sichuan University, Ministry of Education, Chengdu, 610041 China; 5 West China School of Pharmacy, Sichuan University, Chengdu, 610041 China; 6 Department of Pediatrics, West China Second University Hospital, Sichuan University, Chengdu, 610041 China; 7 Laboratory of Molecular Translational Medicine, Center for Translational Medicine, Sichuan University, Chengdu, 610041 China; 8 College of Computer Science, Sichuan University, Chengdu, 610041 China; 9 West China Biomedical Big Data Center, West China Hospital, Sichuan University, Chengdu, 610041 China; 10 Medical Big Data Center, Sichuan University, Chengdu, 610041 China

**Keywords:** Liver injury, Children, Signal analysis, Pharmacovigilance

## Abstract

**Background:**

Evidence of drug-induced liver injury is abundant in adults but is lacking in children. Our aim was to identify suspected drug signals associated with pediatric liver injury.

**Methods:**

Hepatic adverse events (HAEs) among children reported in the Food and Drug Administration Adverse Event Reporting System were analyzed. A descriptive analysis was performed to summarize pediatric HAEs, and a disproportionality analysis was conducted by evaluating reporting odds ratios (RORs) and proportional reporting ratios to detect suspected drugs.

**Results:**

Here, 14,143 pediatric cases were reported, specifically 49.6% in males, 45.1% in females, and 5.2% unknown. Most patients (68.8%) were 6–18 years old. Hospitalization ranked first among definite outcomes (7,207 cases, 37.2%). In total, 264 disproportionate drug signals were identified. The top 10 drugs by the number of reports were paracetamol (1,365; ROR, 3.6; 95% confidence interval (CI), 3.4–3.8), methotrexate (878; ROR, 2.5; 95% CI, 2.3–2.7), vincristine (649; ROR, 3.0; 95% CI, 2.8–3.3), valproic acid (511; ROR, 3.2; 95% CI, 2.9–3.6), cyclophosphamide (490; ROR, 2.4; 95% CI, 2.2–2.6), tacrolimus (427; ROR, 2.4; 95% CI, 2.2–2.7), prednisone (416; ROR, 2.1; 95% CI, 1.9–2.3), prednisolone (401; ROR, 2.3; 95% CI, 2.1–2.5), etoposide (378; ROR, 2.3; 95% CI, 2.1–2.6), and cytarabine (344; ROR, 2.8; 95% CI, 2.5–3.2). After excluding validated hepatotoxic drugs, six were newly detected, specifically acetylcysteine, thiopental, temazepam, nefopam, primaquine, and pyrimethamine.

**Conclusions:**

The hepatotoxic risk associated with 264 signals needs to be noted in practice. The causality of hepatotoxicity and mechanism among new signals should be verified with preclinical and clinical studies.

**Supplementary Information:**

The online version contains supplementary material available at 10.1186/s12887-023-04097-9.

## Background

Drug-induced liver injury (DILI) refers to liver injury caused by the direct toxicity of a drug or its metabolites or by idiosyncratic reactions after exposure to drugs [1–3]. More than 1,100 drugs have been confirmed to be hepatotoxic worldwide, including non-steroidal anti-inflammatory drugs, anti-infective drugs (including anti-tuberculosis drugs), anti-tumor drugs, cardiovascular system drugs, and biological agents [3]. The annual incidence of DILI is estimated to be approximately 23.80 per 100,000 in the general population of China and 12 per 100,000 in Korea [4, 5]. In a prospective study in the US, 11.1% of cases of acute liver failure (ALF) were attributed to DILI [6]. DILI is also an important cause of failures in pharmaceutical research and development, black box warnings added to post-marketing drugs, and drug withdrawals [7].

Children are vulnerable to hepatotoxicity owing to the immaturity of their liver, lack of exclusive drugs, shortage of research data, and poor self-reporting [8–12]. A multicenter study conducted 17 years before this study showed that DILI accounts for 19% of ALF cases in children and that it is the leading cause of pediatric ALF [13]. It is thus essential to conduct studies on pediatric DILI to prevent potential risks for children. In one study, data from 2000 to 2006 were analyzed based on VigiBase and hepatotoxic drugs were compared between children and adults, this information seems insufficient to guide current practice as pediatric medications have been markedly changed [8]. From the implementation of the *Pediatric Research Equity Act* in 2003 to the end of 2018, the Food and Drug Administration (FDA) has accepted over 500 changes to drug labels, most of which expand the population to children [14]. Moreover, most current evidence of pediatric DILI still focuses on case series, and studies based on a large sample size are scarce. This study aimed to characterize hepatic adverse events (HAEs) in children based on the Food and Drug Administration’s Adverse Event Reporting System (FAERS) and to detect suspected drugs associated with liver injury.

## Methods

### Data source

We collected HAEs that might be associated with drugs among people under 18 years of age from 2004Q1 to 2020Q2 in FAERS. FAERS is a large database that supports the FDA’s post-marketing surveillance program for drugs and therapeutic biologicals and has been publicly accessible since 2004 [15]. This database contains adverse event (AE) reports, medication error reports, and product quality complaints submitted by healthcare professionals, consumers, and manufacturers worldwide. For consistency among heterogeneous reporters, all AEs in FAERS were encoded based on the Medical Dictionary for Regulatory Activities (MedDRA) [15]. With a magnitude of reports from the real world, FAERS has the advantage of being widely utilized to detect pharmacovigilant signals and support safe medication in clinical practice [16–18].

### Procedures

In this study, we adopted OpenVigil 2.1 to query liver injury reports from FAERS. OpenVigil 2.1 is a publicly-available and validated tool used for extracting, cleaning, mining, and analyzing data from FAERS [19]. The advantages of this tool, such as being free of charge, its use of clean data, and its convenience for precise results, have made it widely applied in pharmacovigilance studies [20–22]. By summarizing terms associated with liver injury, which might be drug-related, from previous literature, MedDRA 21.1, and standardized MedDRA queries [23–25], we completed our query (Supplementary Table 1). We then unified the drug names into generic names, removed duplicate records, and excluded non-drug reports (iron, aluminum hydroxide, amino acids, etc.).

### Statistical analysis

A descriptive analysis was performed to summarize all pediatric HAEs in terms of age, sex, country, and outcomes. Disproportionality analysis was conducted to quantitatively assess the association between drugs and target AEs by calculating the ratio of target AEs to other AEs in a database and providing the putative relevance from a statistical perspective, which has been extensively applied in pharmacovigilance to evaluate drug–event or vaccine–event relationships for decades [26–28]. We completed a disproportionality analysis based on a 2 × 2 contingency table (Supplementary Table 2), calculated the reporting odds ratio (ROR) and proportional reporting ratio (PRR), and identified a significantly positive drug signal when both the ROR and PRR were greater than the cut-off values (Supplementary Table 3) [29–31].

All statistical analyses were completed using SPSS 26.0 and Excel 2016. We sorted suspected drugs according to the World Health Organization anatomical therapeutic chemical (ATC) classification and reviewed prescribing information and published studies to determine whether HAEs had been verified and whether the drug was metabolized via the liver. The results were ranked according to the number of reports and the ROR.

## Results

### Descriptive analysis

**Fig. 1:**
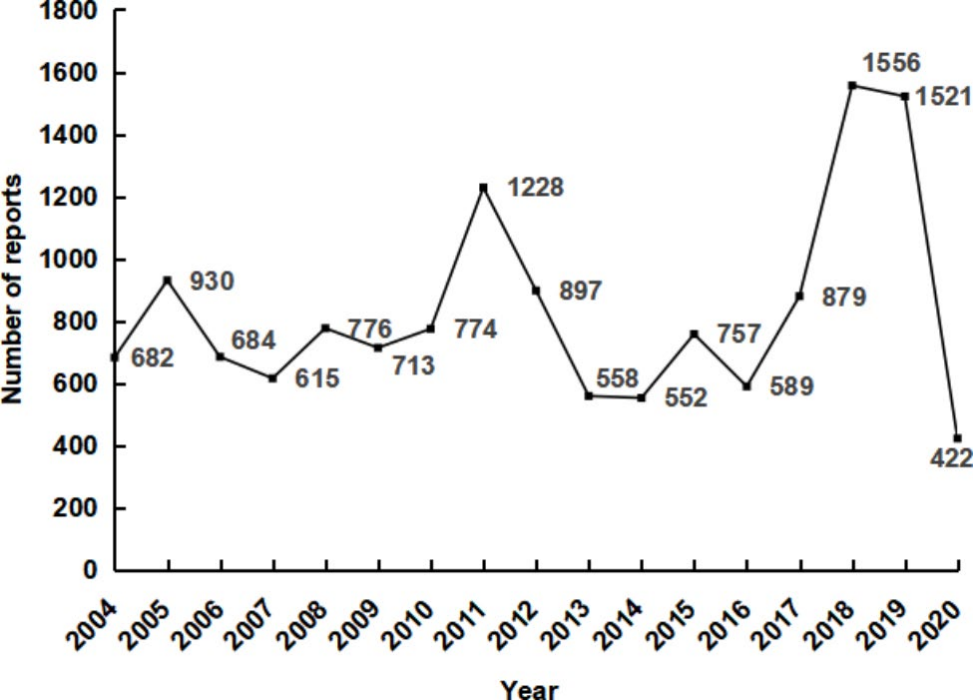
Annual cases of hepatic adverse events in children from 2004 to 2020

From 2004 to 2020, 14,143 pediatric cases of suspected liver injury were submitted to FAERS, of which 49.6% were males, 45.1% were females, and 5.2% were of unknown sex (Table 1). There were 2,358 cases (16.7%) aged 0–2 years, 2,048 cases (45.1%) aged 3–5 years, and 9,737 cases (68.8%) aged 6–18 years. More than 90 countries contributed reports during 2004–2020, mainly in North America and Europe (Supplementary Table 4). The US (5,073 cases, 35.9%), Japan (1,029 cases, 7.3%), France (1,013 cases, 7.2%), the United Kingdom (UK) (964 cases, 6.8%), and Germany (531 cases, 3.8%) were ranked as the top 5 based on the number of reports. The annual number of HAEs in children in 2004–2020 ranged from 422 to 1,556. Peaks were reported in 2018 and 2019 (Fig. 1). The definite outcomes were high-risk, predominantly hospitalization, with 7,207 cases (37.2%) reported, followed by death, life threatening, disability, congenital anomaly, required intervention, and generating injury.

**Table 1:** Characteristics of hepatic adverse events in children in the period 2004–2020

Characteristics	Total reports, n (%)
Sex	
Male	7018 (49.6)
Female	6385 (45.2)
Missing or unknown	740 (5.2)
Age (years)	
0–2	2358 (16.7)
3–5	2048 (14.5)
6–18	9737 (68.8)
Country (top five)	
US	5073 (35.8)
Japan	1029 (7.3)
France	1013 (7.2)
Great Britain	964 (6.8)
Germany	531 (3.8)
Outcome	
Hospitalization	7207 (37.2)
Death	1878 (9.7)
Life-threatening	1535 (7.9)
Disability	448 (2.3)
Congenital anomaly	227 (1.2)
Required intervention	162 (0.8)
Generating injury	162 (0.8)
Other serious or missing outcomes	7758 (40.0)

### Suspected drug signals associated with liver injury in children

We found 2,496 reports, with 586 positive signals suspected to cause liver injury. After unifying the generic names, excluding duplicates, and eliminating non-drug signals, 264 disproportional signals were obtained. The top 10 drugs based on the number of liver injury reports in children were paracetamol (1,365), methotrexate (878), vincristine (649), valproic acid (511), cyclophosphamide (490), tacrolimus (427), prednisone (416), prednisolone (401), etoposide (378), and cytarabine (344), as listed in Table 2. The top 10 drugs by ROR were teceleukin (ROR, 179.8; 95% confidence interval (CI), 22.5–1,437.4), cefpirome (ROR, 67.4; 95% CI, 7.0–647.8), trabectedin (ROR, 56.2; 95% CI, 10.9–289.5), ciprofloxacin (ROR, 44.9; 95% CI, 11.2–179.7), propoxyphene napsylate (ROR, 44.9; 95% CI, 8.2–245.3), vandetanib (ROR, 35.3; 95% CI, 13.7–91.1), calcium levofolinate (ROR, 33.7; 95% CI, 5.6–201.6), valsartan and amlodipine (ROR, 25.3; 95% CI, 9.8–65.5), aclarubicin (ROR, 22.5; 95% CI, 4.5–111.3), and sovaprevir (ROR, 22.5; 95% CI, 10.7–47.2), as shown in Table 3.

**Table 2:** Top 10 drugs by number of hepatic injury reports in children

Drug (ATC code-5th level)	Number of reports	PRR	χ²	ROR (95% CI)
Paracetamol (N02BE01)	1365	3.3	1,968.2	3.6 (3.4–3.8)
Methotrexate (L01BA01)	878	2.4	668.6	2.5 (2.3–2.7)
Vincristine (L01CA02)	649	2.8	728.4	3.0 (2.8–3.3)
Valproic acid (N03AG01)	511	3	661.3	3.2 (2.9–3.6)
Cyclophosphamide (L01AA01)	490	2.3	342	2.4 (2.2–2.6)
Tacrolimus (L04AD02)	427	2.3	310.2	2.4 (2.2–2.7)
Prednisone (H02AB07)	416	2	211.8	2.1 (1.9–2.3)
Prednisolone (H02AB06)	401	2.2	256.3	2.3 (2.1–2.5)
Etoposide (L01CB01)	378	2.2	249.6	2.3 (2.1–2.6)
Cytarabine (L01BC01)	344	2.6	349.4	2.8 (2.5–3.2)

ATC, anatomic therapeutic chemical; CI, confidence interval; PRR, proportional reporting ratio; ROR, reporting odds ratio

**Table 3:** Top 10 drugs in terms of ROR in children

Drug (ATC code-5th level)	Number of reports	PRR	χ²	ROR (95% CI)
Teceleukin (NA)	8	20.9	137.9	179.8 (22.5–1,437.4)
Cefpirome (J01DE02)	3	17.6	33.2	67.4 (7.0–647.8)
Trabectedin (L01CX01)	5	16.8	61.8	56.2 (10.9–289.5)
Ciprofloxacin (J01MA02)	6	15.6	71.3	44.9 (11.2–179.7)
Propoxyphene napsylate (NA)	4	15.6	43	44.9 (8.2–245.3)
Vandetanib (L01XE12)	11	14.3	128.9	35.3 (13.7–91.1)
Calcium levofolinate (V03AF04)	3	14.1	25.6	33.7 (5.6–201.6)
Valsartan and amlodipine (C09DB01)	9	12.4	87.1	25.3 (9.8–65.5)
Aclarubicin (L01DB04)	3	11.7	20.6	22.5 (4.5–111.3)
Sovaprevir (M09AX09)	14	11.7	132.5	22.5 (10.7–47.2)

ATC, anatomic therapeutic chemical; CI, confidence interval; PRR, proportional reporting ratio; ROR, reporting odds ratio

The 264 positive signals were then classified according to the ATC 1st level class, that is, the anatomical class, as shown in Table 4. Anti-infectives for systemic use were the most reported class, comprising 75 suspected drugs, followed by anti-neoplastic and immunomodulatory agents, with 69 signals. Each drug was checked to determine whether hepatic injury information had been provided on the package inserts and whether the drug was metabolized via the liver. The details of all positive signals are listed in Supplementary Table 5. After excluding drugs with hepatotoxic information provided in the package inserts or declared in published studies, six of 264 were identified, all of which were disproportionately associated with liver injury in children, namely acetylcysteine, thiopental, temazepam, nefopam, primaquine, and pyrimethamine (Table 5).

**Table 4:** Suspected drugs associated with liver injury in children by anatomical class

ATC code (1st level)	Anatomical main group		Number of suspected drugs, n (%)
J	Anti-infectives for systemic use		75 (28.4)
L	Antineoplastic and immunomodulating agents		69 (26.1)
N	Nervous system		26 (9.8)
A	Alimentary tract and metabolism		20 (7.6)
C	Cardiovascular system		17 (6.4)
M	Musculo-skeletal system		10 (3.8)
B	Blood and blood-forming organs		8 (3.0)
P	Antiparasitic products, insecticides, and repellents		7 (2.7)
H	Systemic hormonal preparations, excl. sex hormones and insulins		6 (2.3)
V	Various		6 (2.3)
R	Respiratory system		4 (1.5)
D	Dermatologicals		2 (0.8)
G	Genito urinary system and sex hormones		1 (0.4)
NA	No ATC code		13 (4.9)
Total		264 (100)	

ATC: anatomic therapeutic chemical

**Table 5:** Suspected drugs associated with liver injury in children but not labeled as such in medicine specifications

Drug (ATC code-5th level)	Number of reports	PRR	χ²	ROR (95% CI)	Metabolized via the liver	Hepatoxicity in prescribing information
Acetylcysteine (R05CB01)	19	2.8	20.3	3.0 (1.9–4.8)	Yes	No
Thiopental (N05CA19)	16	3.1	22	3.5 (2.0–5.9)	Yes	No
Temazepam (N05CD07)	9	2.2	5.3	2.4 (1.2–4.7)	Yes	No
Nefopam (N02BG06)	5	10.7	36.2	18.7 (5.7–61.3)	Yes	No
Primaquine (P01BA03)	5	9	29.3	14.0 (4.6–42.9)	Yes	No
Pyrimethamine (P01BD01)	4	3.1	4	3.5 (1.2–9.9)	Yes	No

ATC, anatomic therapeutic chemical classification; CI, confidence interval; PRR, proportional reporting ratio; ROR, reporting odds ratio

## Discussion

In this study, suspected drug signals related to pediatric liver injury were identified by conducting a disproportional analysis of data from FAERS in the period 2004–2020 to provide information on safe medication for children. To the best of our knowledge, this study confirms the issues raised in the previous literature and provides new insights into pediatric liver injury associated with drugs. In 2010, Ferrajolo et al. explored drug-induced hepatic injury in children based on VigiBase from 2000 to 2006 in a case/non-case study [8]. Both paracetamol and methotrexate were among the top 10 drugs according to the number of reports in previous and current studies. Forty-two suspected drugs identified in the former (e.g., basiliximab, caspofungin, isoniazid) were also identified in our results. Basiliximab, a novel selective anti-human interleukin-2 receptor monoclonal antibody used for immunosuppressive therapy, was the only novel signal detected in children in a previous study, which suggested future monitoring, and this was confirmed herein. Liver injury associated with basiliximab has not been included in the FDA’s prescribing information, but the incidence of abnormal liver biochemical parameters associated with basiliximab, which is greater than 5%, has been indicated by Japanese package inserts [32, 33]. A randomized controlled trial of adults found an increase in serum alkaline phosphatase within the first month after the administration of basiliximab, but it was considered to be “functional cholestasis” [34, 35]. No elevated liver enzyme levels or hepatotoxicity were reported in clinical trials among children treated with basiliximab [36, 37].

Forty-three signals found in Ferrajolo’s study (chlorprothixene, methylphenidate, infliximab, etc.) were not included here. The reasons for this could be as follows. (1) Different data sources. VigiBase is the world’s largest database of individual case safety reports, having collected data on AEs from more than 130 countries since the late 1960s, whereas FAERS has collected data reported to the FDA only since 2004 [38]. (2) Distinct algorithms have been used for signal detection. Only ROR was adopted in the former, whereas ROR and PRR were combined here. (3) Some drugs were withdrawn from the market in their early days and their AE reporting ratios were diluted. Compared with the 2010 study, 222 suspicious drug signals were newly detected in the current study, including drugs marketed before 2006, such as ganciclovir and amlodipine, and after 2006, such as vandetanib and gemtuzumab.

Anti-infectives, especially antibacterial drugs, are extensively applied, and their irrational use can cause hepatotoxicity. The immature liver function in children and increased hepatotoxicity caused by the concomitant administration of multiple antibacterial drugs make children more vulnerable to hepatic injury than adults. The results of anti-infectives for systemic use, as the most reported class in our study, were consistent with the published literature [39, 40]. Representative drugs included trimethoprim-sulfamethoxazole (TMP-SMX), ceftriaxone, fluconazole, and isoniazid.

TMP-SMX exerts its antimicrobial effect by blocking the metabolism of folic acid through a dual pathway and can cause DILI via allergic reactions (fever, rash, etc.) [41]. Bell et al. [42] reported a case of DILI induced by TMP-SMX; specifically, a 9-year-old boy with a community-acquired methicillin-resistant *Staphylococcus aureus* skin and soft tissue infection received TMP-SMX, and 14 days after administration, he developed fever, vomiting, and abdominal pain, with poor appetite and mentality. He was diagnosed with TMP-SMX-induced liver injury based on biochemical tests and the exclusion of other factors contributing to hepatic injury.

Ceftriaxone is a semi-synthetic, third-generation cephalosporin that mainly causes cholelithiasis or bile stasis [43]. One retrospective study showed that 3.2% of patients treated with ceftriaxone might develop liver injury [44]. Liver injury caused by ketoconazole in children mostly presents as elevated levels of direct bilirubin and liver enzymes [45]. Liver functions usually recover after drug cessation, but severe cases can also develop into liver failure [46, 47]. Animal studies have confirmed the dose-dependent liver injury induced by ketoconazole [48].

Isoniazid is a first-line anti-tuberculosis drug. Approximately 10–20% of patients administered isoniazid develop the transient elevation of alanine aminotransferase, and fewer than 1–3% develop severe liver injury or even liver failure [49, 50]. A greater risk of isoniazid- and rifampicin-related liver injury in children (6.9% in children and 2.7% in adults) was reported in a retrospective study [51]. In the US, the incidence of isoniazid-associated hepatotoxicity was determined to be 1% in children treated for latent tuberculosis infection, and there was a synergistic harmful effect on the liver when isoniazid and rifampin were used in one prescription [52, 53]. It is generally believed that isoniazid-induced liver injury is attributed to the toxicity of the metabolites acetylhydrazine and hydrazine [54]. Moreover, mitochondrial damage caused by isoniazid-induced oxidative stress and lipid peroxidation stimulated by isoniazid and its metabolites has also been discussed [55].

In this study, six signals were found to be disproportionally associated with pediatric liver injury for the first time, namely acetylcysteine, thiopental, temazepam, nefopam, primaquine, and pyrimethamine. Acetylcysteine (also known as N-acetylcysteine, NAC) was detected as a disproportional signal associated with pediatric hepatic injury (ROR, 3.0; 95% CI 1.9–4.8). None of these HAEs had been reported in either package inserts or the published literature since NAC was marketed. NAC is the only drug approved by the FDA for the treatment of DILI caused by acetaminophen (APAP) overdose and might prevent hepatic injury by restoring glutathione levels [56]. A dosage of NAC for children greater than 5 kg is set based on clinical practice; however, the safety and effectiveness of NAC have not been verified in adequate and well-controlled studies. The *ACG Clinical Guideline: Diagnosis and Management of Idiosyncratic Drug-Induced Liver Injury* suggests the use of NAC for children with ALF caused by severe DILI, as a significantly lower 1-year spontaneous survival rate was associated with the intravenous infusion of NAC in children with non-APAP ALF in a placebo-controlled clinical trial [2, 57]. In our study, we found that NAC was a secondary suspected or concomitant drug in most reports and a primary suspicious drug in one report where APAP was secondary. Therefore, as a disproportional signal, NAC is used to treat or prevent HAEs, especially APAP-induced HAEs. However, whether NAC has a negative effect on children with non-APAP ALF is unclear.

We also found an association between temazepam use and pediatric liver injury (ROR, 2.4; 95% CI, 1.2–4.7). No hepatotoxic risk is indicated in the prescribing information, and there is a lack of evidence to ensure the effectiveness and safety of temazepam among children. Temazepam is a benzodiazepine, and it is uncommon to observe elevated hepatic enzymes with benzodiazepine used and to report hepatoxic cases in practice. However, it has been reported that benzodiazepines (alprazolam, chlordiazepoxide, clonazepam, diazepam, flurazepam, and triazolam) are associated with rare cholestatic liver injuries [58, 59]. We have not retrieved any literature directly reporting temazepam-induced liver injury and postulated that this is due to its lower frequency of medication and shorter duration [2]. In addition, there is a possibility that cross-sensitivity to temazepam might occur with other benzodiazepines [58].

In our study, nefopam was disproportionally associated with liver injury in children (ROR, 18.7; 95% CI, 5.7–61.3). It is unclear whether nefopam is effective and safe for children as a painkiller. In this study, we identified five nefopam-related pediatric hepatotoxic AEs. Four patients reported nefopam as a secondary suspected drug, and one reported nefopam as a concomitant drug. The primary suspected drugs in these reports were ketoprofen, esomeprazole, and pregabalin, all of which have adverse effects on the liver, as specified in the package inserts. Whether it is safe to use nefopam alone in children merits attention in clinical practice.

In this study, thiopental was detected as a disproportional signal (ROR, 3.5; 95% CI, 2.0–5.9). Thiopental is an intravenous anesthetic without hepatotoxic information provided in the inserts. In our findings, most reports of HAEs in children associated with thiopental recorded it as a concomitant drug, and the suspected drugs were mainly propofol, lamotrigine, phenytoin, propranolol, valproic acid, carbamazepine, lacosamide, and clonazepam, all of which have hepatotoxicity labeled in their package inserts. The risk of hepatotoxicity associated with this drug in children when applied alone is unclear. Bedir et al. indicated that oxidative stress and inflammation develop in the liver tissue of rats injected with thiopental alone, but further clinical studies are needed to explore this effect in humans [60].

Pyrimethamine was also detected as a disproportional signal (ROR, 3.5; 95% CI, 1.2–9.9). Four reports of pyrimethamine-associated hepatic injury in children have been identified. One patient recorded pyrimethamine as the primary suspected drug and sulfadiazine as the secondary drug, of which liver injury was a definite adverse effect. In the other three reports, the primary suspected drugs associated with liver injury were clindamycin, calcium folinate, and isotretinoin, all of which were reported to have a risk of hepatotoxicity. Pyrimethamine is typically combined with sulfonamides in practice. Many case reports of liver injury, such as granulomatous hepatitis, hypersensitivity to liver injury, and fatal hepatic necrosis, were determined to be induced by pyrimethamine-sulfadoxine [61–63]. Whether pyrimethamine alone can cause liver injury requires further investigation.

We found that primaquine is associated with pediatric HAEs (ROR, 14.0; 95% CI, 4.6–42.9). Among the five liver injury records associated with primaquine, one reported primaquine as the primary suspected drug, and four reported primaquine as a secondary suspected or concomitant drug, with chloroquine, tocilizumab, and malarone as the primary suspected drugs, all of which are toxic to the liver. The safety of primaquine when applied alone in children is not clear, as current studies have only presented an elevation of biochemical indicators of the liver when primaquine is used with chloroquine [64, 65].

This study has both limitations and strengths. Firstly, due to the differences in physiological characteristics, biochemical parameters, and disease spectrum between children and adults, and the lack of prospective data on cases in FAERS, such as viral antibody tests and re-exposure results, it is not appropriate to use tools that aid in the diagnosis of DILI to establish a robust causal relationship indicating that a drug ‘caused’ the liver injury in children. For example, the Roussel-Uclaf Causality Assessment Method is the most commonly used tool to assess DILI in adults, but it is not available as an independent assessment for children and is more appropriate for prospective data [66]. In this study, the association between suspected drugs and HAEs was derived from a statistical perspective. Secondly, HAEs in children may be under-reported due to the wide variation in the clinical presentation of HAEs, ranging from asymptomatic elevated liver enzymes to ALF, and the weak autonomic expression in children. For example, specific hepatotoxic drugs for children may be ignored if liver injury is not manifest. Despite its limitations, this study has the following non-negligible advantages. Firstly, this study uses a robustly validated and easily accessible real-world database, combined with advanced tools and commonly recommended pharmacovigilance methods, to statistically detect the association between drugs and HAEs in children, and to supplement clinical information with prescribing information and published literature. Secondly, unlike previous studies in children with small sample sizes, such as case reports and case series, this study reports the characteristics of HAEs and suspected drugs in a specific population of children based on a large volume of data (~ 14,000 cases) spanning a long time period (2004–2020), which can provide information for clinical drug safety in children and guide future independent studies in this important area of DILI for the pediatric population.

## Conclusion

In our study, HAEs were analyzed in children from 2004 to 2020 based on FAERS, and suspected drug signals were discovered. In total, 264 drugs were identified as having a disproportional association with pediatric liver injury, six of which were detected for the first time. These findings call for attention in clinical practice to reduce potential risks, and preclinical and clinical studies are expected to verify the signals, determine the hepatotoxic mechanism, and promote the safe use of medication in pediatric patients.

### Electronic supplementary material

Below is the link to the electronic supplementary material.

Supplementary Material 1

## Data Availability

The authors confirm the data and materials supporting the findings of this study are available within the article and its supplementary materials. FAERS reports are publicly available on the FDA website (https://fis.fda.gov/extensions/FPD-QDE-FAERS/FPD-QDE-FAERS.html).

## List of abbreviations

AE: Adverse event
ALF: Acute liver failure
APAP: Acetaminophen
ATC: Anatomical therapeutic chemical
DILI: Drug-induced liver injury
FAERS: Food and Drug Administration’s Adverse Event Reporting System
FDA: Food and Drug Administration
HAEs: Hepatic adverse events
MedDRA: Medical Dictionary for Regulatory Activities
NAC: N-acetylcysteine
PRR: Proportional reporting ratio
ROR: Reporting odds ratio
TMP-SMX: Trimethoprim-sulfamethoxazole
