# Supplementary material for: Liver injury in children: signal analysis of suspected drugs based on the food and drug administration adverse event reporting system
Source: BMC Pediatr. 2023 Sep 28;23:492. doi: 10.1186/s12887-023-04097-9 (PMC10537493; doi:10.1186/s12887-023-04097-9)
Supplement: Supplementary file 1 — Supplementary Material 1 [file 12887_2023_4097_MOESM1_ESM.pdf]

Supplementary Table 1 Terms of drug-related hepatic injury

PT	
Acute hepatic failure	Hepatic necrosis
Alanine aminotransferase abnormal	Hepatitis
Alanine aminotransferase increased	Hepatitis acute
Aspartate aminotransferase abnormal	Hepatitis cholestatic
Aspartate aminotransferase increased	Hepatitis fulminant
Bilirubin conjugated increased	Hepatitis toxic
Bilirubin urine	Hepatotoxicity
Blood bilirubin abnormal	Hyperbilirubinaemia
Blood bilirubin increased	Jaundice
Blood bilirubin unconjugated increased	Jaundice cholestatic
Cholestasis	Jaundice hepatocellular
Coma hepatic	Liver function test abnormal
Cytolytic hepatitis	Liver injury
Drug-induced liver injury	Liver transplant
Hepatocellular injury	Mixed liver injury
Hepatic encephalopathy	Subacute hepatic failure
Hepatic enzyme abnormal	Transaminases abnormal
Hepatic enzyme increased	Transaminases increased
Hepatic failure	Urine bilirubin increased
Hepatic function abnormal	
**SMQ**	
Drug related hepatic disorders - comprehensive search	Drug related hepatic disorders - severe events only

PT: preferred terms, SMQ: standardized Medical Dictionary for Regulatory Activities query

Supplementary Table 2 2×2 contingency table for disproportionality analysis

Drugs/AEs	AEs of interest	Other AEs	Sums
Drug of interest	a	b	a+b
Other drugs	c	d	c+d
Sums	a+c	b+d	n

AEs: adverse events

Supplementary Table 3 Formulas and thresholds for a positive signal

Algorithms	Formula	Cut-offs for a positive signal
ROR	$\mathrm{ROR}=\frac{a/c}{b/d}$<br>95% CI= $e^{\ln(\mathrm{ROR})\pm 1.96\sqrt{\frac{1}{a}+\frac{1}{b}+\frac{1}{c}+\frac{1}{d}}}$	①a ≥3；<br>②The low limit of 95% CI >1
PRR	$\mathrm{PRR}=\frac{a/(a+b)}{c/(c+d)}$<br>$\chi^2=\frac{(\lvert ad-bc\rvert-n/2)^2 n}{(a+b)(a+c)(c+d)(b+d)}$	①a ≥ 3；<br>②PRR ≥ 2；<br>③χ^2≥ 4.

CI: confidence interval, PRR: proportional reporting ratio, ROR, reporting odds ratio

Supplementary Table 4 Countries reporting suspected liver injury of children in the FAERS database from 2004-2020

No.	Country	Reports, n (%)	No.	Country	Reports, n (%)
1	United States	5,072(35.9)	49	Uganda	13(0.1)
2	Japan	1,029(7.3)	50	Russia	12(0.1)
3	France	1,013(7.2)	51	Slovenia	12(0.1)
4	United Kingdom	964(6.8)	52	Peru	11(0.1)
5	Germany	531(3.8)	53	Croatia	10(0.1)
6	Canada	412(2.9)	54	Venezuela	10(0.1)
7	Italy	368(2.6)	55	Lebanon	9(0.1)
8	Spain	324(2.3)	56	Nigeria	9(0.1)
9	Turkey	260(1.8)	57	Arab	9(0.1)
10	China	313(2.2)	58	Costa Rica	8(0.1)
11	India	205(1.4)	59	Pakistan	8(0.1)
12	Australia	145(1)	60	Serbia	8(0.1)
13	Poland	134(0.9)	61	Jordan	7(0)
14	Brazil	123(0.9)	62	Botswana	6(0)
15	Netherlands	120(0.8)	63	Cyprus	6(0)
16	Portugal	85(0.6)	64	Ukraine	6(0)
17	South Africa	84(0.6)	65	Bulgaria	5(0)
18	Switzerland	82(0.6)	66	Albania	4(0)
19	North Korea	65(0.5)	67	Congo	4(0)
20	New Zealand	63(0.4)	68	Indonesia	4(0)
21	Mexico	60(0.4)	69	Kazakhstan	4(0)
22	Denmark	56(0.4)	70	Kuwait	4(0)
23	Belgium	55(0.4)	71	Malawi	4(0)
24	Israel	53(0.4)	72	Morocco	4(0)
25	Argentina	50(0.4)	73	Vietnam	4(0)
26	Colombia	50(0.4)	74	Ghana	3(0)
27	Thailand	48(0.3)	75	Qatar	3(0)
28	Greece	45(0.3)	76	Côte d'Ivoire	2(0)
29	Romania	37(0.3)	77	Bolivia	2(0)
30	Russia	36(0.3)	78	Iraq	2(0)
31	Ireland	32(0.2)	79	Kenya	2(0)
32	Norway	32(0.2)	80	Latvia	2(0)
33	Finland	31(0.2)	81	Senegal	2(0)
34	Austria	29(0.2)	82	Sri Lanka	2(0)
35	Hungary	29(0.2)	83	Swaziland	2(0)
36	Chile	26(0.2)	84	Libya	2(0)
37	Czech Republic	26(0.2)	85	Bangladesh	1(0)
38	Slovakia	25(0.2)	86	Cameroon	1(0)
39	Algeria	23(0.2)	87	Cuba	1(0)
40	Singapore	23(0.2)	88	East Timor	1(0)
41	Tunisia	22(0.2)	89	Estonia	1(0)
42	Egypt	21(0.1)	90	Lithuania	1(0)
43	Iran	20(0.1)	91	Malta	1(0)
44	Oman	19(0.1)	92	Moldova	1(0)
45	Iceland	18(0.1)	93	Tajikistan	1(0)
46	Malaysia	18(0.1)	94	Uruguay	1(0)
47	Saudi Arabia	18(0.1)	95	Missing or unknown	1,550(11)
48	Philippines	14(0.1)			

FAERS: Food and Drug Administration's Adverse Event Reporting System

Supplementary Table 5 All positive signals of pediatric hepatic AEs

ATC code (2nd level)	Drug (ATC code-5th level)	Number of reports	PRR	χ^2	ROR（95%CI）	Metabolized via the liver	hepatic AEs in prescribing information
A02	Rebamipide (A02BX14)	6	6.7	24.7	9.0 (3.5-23.2)	Yes	Yes
	Famotidine (A02BA03)	4	3.1	4	3.5 (1.2-9.9)	Yes	Yes
	Omeprazole (A02BC01)	4	4.5	7.9	5.3 (1.8-15.7)	Yes	Yes
	Lansoprazole (A02BC03)	3	8.8	14.3	13.5 (3.2-56.4)	Yes	Yes
A03	Mosapride citrate (A03FA09)	3	5.9	8.1	7.5 (2.0-27.7)	Yes	Yes
A04	Granisetron (A04AA02)	14	2.8	15.2	3.1 (1.7-5.3)	Yes	Yes
	Flupirtine (A04AD12)	5	4.7	11.6	5.6 (2.1-15.0)	Yes	Yes
A05	Ursodeoxycholic acid (A05AA02)	69	4.3	180.4	5.1 (3.9-6.6)	Yes	Yes
A07	Amphotericin B (A07AA07)	77	2.2	49.7	2.3 (1.8-2.9)	Yes	Yes
	Loperamide (A07DA03)	37	2.2	22.9	2.3 (1.6-3.2)	Yes	Yes
	Sulfasalazine (A07EC01)	27	2.3	20	2.5 (1.7-3.7)	Yes	Yes
A10	Pioglitazone (A10BG03)	6	3.8	10.2	4.3 (1.8-10.4)	Yes	Yes
A11	Alfacalcidol (A11CC03)	15	2.9	18	3.2 (1.9-5.5)	Yes	Yes
	Nicotinamide (A11HA01)	11	3.5	17.9	3.9 (2.1-7.4)	Yes	Yes
	Pyridoxal phosphate (A11HA06)	8	4.5	19	5.3 (2.4-11.4)	Yes	Yes
A16	Nitisinone (A16AX04)	105	11.8	1059.8	22.4 (17.1-29.4)	No	Yes
	Glycerol phenylbutyrate (A16AX09)	104	8.9	747.7	13.7 (10.7-17.5)	Yes	Yes
	Caglutamic acid (A16AA05)	31	5.4	110	6.6 (4.4-9.9)	Yes	Yes
	Asfotase alpha (A16AB13)	27	3.1	38.1	3.4 (2.3-5.1)	No	Yes
	Sodium benzoate (A16AX11)	11	7.2	54.7	9.9 (4.9-20.1)	Yes	Yes
B01	Warfarin (B01AA03)	142	3.7	288.2	4.2 (3.5-5.1)	Yes	Yes
	Enoxaparin (B01AB05)	51	2.2	32.2	2.3 (1.7-3.1)	Yes	Yes
	Dapoxetine (B01AB04)	10	4	20.9	4.7 (2.4-9.3)	Yes	Yes
	Apixaban (B01AF02)	8	2.7	7.1	2.9 (1.4-6.1)	Yes	Yes
	Beraprost (B01AC19)	7	3.7	11.4	4.1 (1.8-9.3)	Yes	Yes
	Fondaparinux sodium (B01AX05)	7	6.6	28.9	8.7 (3.6-20.9)	Yes	Yes
	Phenprocoumon (B01AA04)	5	4.7	11.6	5.6 (2.1-15.0)	Yes	Yes
B02	Phytomenadione (B02BA01)	34	5.1	112.2	6.2 (4.3-9.1)	Yes	Yes
C01	Digoxin (C01AA05)	45	2.3	32.7	2.4 (1.8-3.3)	Yes	Yes
	Amiodarone (C01BD01)	30	2.4	24.4	2.6 (1.8-3.8)	Yes	Yes
	Milrinone (C01CE02)	13	2.2	7.9	2.4 (1.3-4.2)	No	Yes
	Propafenone (C01BC03)	12	2.8	12.3	3.0 (1.6-5.5)	Yes	Yes

C02	Bosentan (C02KX01)	185	2	98.6	2.1 (1.8-2.5)	Yes	Yes
	Macitentan (C02KX04)	24	2.1	14.1	2.3 (1.5-3.4)	Yes	Yes
	Hydralazine (C02DB02)	18	4.7	50.8	5.6 (3.4-9.4)	Yes	Yes
C03	Furosemide (C03CA01)	152	2	80.7	2.1 (1.8-2.5)	Yes	Yes
	Spironolactone (C03DA01)	75	2.2	51	2.3 (1.9-3.0)	Yes	Yes
	Tolvaptan (C03XA01)	10	6.2	40	8.0 (3.9-16.5)	Yes	Yes
C07	Carvedilol (C07AG02)	19	2.3	13.2	2.4 (1.5-3.9)	Yes	Yes
C08	Amlodipine (C08CA01)	10	2.4	7.1	2.6 (1.3-4.9)	Yes	Yes
C09	Captopril (C09AA01)	29	2.2	19	2.3 (1.6-3.4)	Yes	Yes
	Valsartan and amlodipine (C09DB01)	9	12.4	87.1	25.3 (9.8-65.5)	Yes	Yes
C10	Fenofibrate (C10AB05)	13	4	27	4.6 (2.5-8.3)	Yes	Yes
	atorvastatin (C10AA05)	7	2.5	4.9	2.6 (1.2-5.7)	Yes	Yes
	Fluvastatin (C10AA04)	3	4.1	4.5	4.8 (1.4-16.7)	Yes	Yes
H01	Octreotide (H01CB02)	18	3.1	24.7	3.4 (2.1-5.6)	Yes	Yes
	Pegvisomant (H01AX01)	6	8.3	32.9	12.3 (4.5-33.1)	No	Yes
H02	Prednisone (H02AB07)	416	2	211.8	2.1 (1.9-2.3)	Yes	Yes
	Prednisolone (H02AB06)	401	2.2	256.3	2.3 (2.1-2.5)	Yes	Yes
	Methylprednisolone (H02AB04)	219	2	111.9	2.1 (1.8-2.4)	Yes	Yes
H03	Propylthiouracil (H03BA02)	8	4.5	19	5.3 (2.4-11.4)	Yes	Yes
J01	Trimethoprim (J01EA01)	336	2.9	417.3	3.1 (2.8-3.5)	Yes	Yes
	Sulfamethoxazole (J01EC01)	291	2.8	344.5	3.1 (2.7-3.5)	Yes	Yes
	Minocycline (J01AA08)	197	4.7	584	5.6 (4.8-6.6)	Yes	Yes
	Ceftriaxone (J01DD04)	162	2.6	167.7	2.8 (2.4-3.4)	Yes	Yes
	Vancomycin (J01XA01)	131	2.3	93.6	2.4 (2.0-2.9)	Yes	Yes
	Sulfamethoxazole and trimethoprim (J01EE01)	126	3.5	225.5	3.9 (3.2-4.7)	Yes	Yes
	Meropenem (J01DH02)	71	2.4	58.6	2.6 (2.0-3.3)	Yes	Yes
	Levofloxacin (J01MA12)	61	3	83	3.3 (2.5-4.3)	Yes	Yes
	Cefotaxime (J01DD01)	59	3.2	90.4	3.5 (2.7-4.7)	Yes	Yes
	Piperacillin (J01CA12)	53	2.5	48	2.7 (2.0-3.6)	No	Yes
	Tazobactam (J01CG02)	50	2.4	41	2.6 (1.9-3.4)	Yes	Yes
	Amikacin (J01GB06)	49	2.7	50.9	2.9 (2.1-3.9)	No	Yes
	Ampicillin (J01CA01)	49	2.2	33.6	2.4 (1.8-3.2)	Yes	Yes
	Linezolid (J01XX08)	44	2.4	35.9	2.6 (1.9-3.5)	Yes	Yes
	Ceftazidime (J01DD02)	34	3.1	47.7	3.4 (2.4-4.9)	No	Yes
	Benzylpenicillin (J01CE01)	29	2.4	22.6	2.5 (1.7-3.7)	Yes	Yes

	Amoxicillin/clavulanic acid potassium (J01CR02)	27	2.1	15.5	2.2 (1.5-3.3)	Yes	Yes
	Moxifloxacin (J01MA14)	24	2.7	24.4	2.9 (1.9-4.4)	Yes	Yes
	Cefepime (J01DE01)	21	2	10.2	2.1 (1.4-3.3)	Yes	Yes
	Teicoplanin (J01XA02)	19	3.3	29.5	3.7 (2.3-6.0)	No	Yes
	Cefazolin (J01DB04)	17	2.5	13.9	2.6 (1.6-4.4)	Yes	Yes
	Streptomycin (J01GA01)	17	4	36.7	4.6 (2.7-7.8)	No	Yes
	Oxacillin (J01CF04)	16	7.7	89.9	10.9 (6.0-19.8)	Yes	Yes
	Ofloxacin (J01MA01)	15	2.8	16.5	3.1 (1.8-5.3)	Yes	Yes
	Piperacillin/tazobactam (J01CR05)	12	3.6	21	4.1 (2.2-7.6)	Yes	Yes
	Sulbactam (J01CG01)	12	2.2	7.1	2.3 (1.3-4.3)	No	Yes
	Cefpodoxime (J01DD13)	11	2.5	8.5	2.6 (1.4-4.9)	No	Yes
	Daptomycin (J01XX09)	10	4.4	24.2	5.2 (2.6-10.4)	No	Yes
	Telithromycin (J01FA15)	8	5.1	23.2	6.2 (2.8-13.6)	Yes	Yes
	Doxycycline hydrochloride (J01AA02)	7	4.6	16.8	5.4 (2.4-12.4)	Yes	Yes
	Phenoxymethylpenicillin (J01CE02)	7	4.6	16.8	5.4 (2.4-12.4)	Yes	Yes
	Ciprofloxacin (J01MA02)	6	15.6	71.3	44.9 (11.2-179.7)	Yes	Yes
	Norfloxacin (J01MA06)	5	5.9	16.3	7.5 (2.7-20.6)	Yes	Yes
	Tigecycline (J01AA12)	3	4.1	4.5	4.8 (1.4-16.7)	Yes	Yes
	Cefpirome (J01DE02)	3	17.6	33.2	67.4 (7.0-647.8)	Yes	Yes
	Sulfadiazine (J01EC02)	3	4.4	5.1	5.2 (1.5-18.2)	Yes	Yes
J02	Fluconazole (J02AC01)	101	2.7	109.4	2.9 (2.4-3.6)	Yes	Yes
	Voriconazole (J02AC03)	92	2.9	116.7	3.2 (2.5-3.9)	Yes	Yes
	Micafungin (J02AX05)	27	2.7	28.3	2.9 (2.0-4.4)	Yes	Yes
	Caspofungin (J02AX04)	12	4	25.4	4.6 (2.5-8.7)	Yes	Yes
	Posaconazole (J02AC04)	6	3.4	8	3.7 (1.6-8.9)	Yes	Yes
J04	Isoniazid (J04AC01)	205	7.2	1106.1	9.8 (8.3-11.5)	Yes	Yes
	Rifampicin (J04AB02)	192	6.4	883.9	8.3 (7.0-9.8)	Yes	Yes
	Pyrazinamide (J04AK01)	141	7.2	768.5	9.9 (8.1-12.1)	Yes	Yes
	Ethambutol (J04AK02)	108	6.7	538.1	9.0 (7.2-11.2)	Yes	Yes
	Dapsone (J04BA02)	27	3.3	42.4	3.7 (2.4-5.5)	Yes	Yes
	Clofazimine (J04BA01)	18	4.5	47.5	5.3 (3.2-8.9)	Yes	Yes
	Bedaquiline (J04AK05)	13	6.6	59.2	8.9 (4.7-16.8)	Yes	Yes
	Ethionamide (J04AD03)	11	3.1	14.7	3.5 (1.8-6.6)	Yes	Yes
	Cycloserine (J04AB01)	10	3.7	18.1	4.2 (2.2-8.3)	Yes	Yes
	Delamanid (J04AK06)	10	5.1	30.3	6.2 (3.1-12.6)	Yes	Yes

	Protionamide (J04AD01)	5	9	29.3	14.0 (4.6-42.9)	Yes	Yes
	Capreomycin (J04AB30)	4	3.6	5.4	4.1 (1.4-11.9)	Yes	Yes
J05	Lamivudine (J05AF05)	166	2.3	122	2.4 (2.1-2.8)	Yes	Yes
	Aciclovir (J05AB01)	111	2.4	89.2	2.5 (2.1-3.1)	Yes	Yes
	Abacavir (J05AF06)	72	3.1	106.8	3.5 (2.7-4.5)	Yes	Yes
	Nevirapine (J05AG01)	66	2.2	41.2	2.3 (1.8-2.9)	Yes	Yes
	Atazanavir (J05AE08)	58	3.6	108.6	4.0 (3.0-5.3)	Yes	Yes
	Didanosine (J05AF02)	58	3.4	101.3	3.8 (2.9-5.1)	Yes	Yes
	Stavudine (J05AF04)	52	2.9	64.6	3.1 (2.4-4.2)	Yes	Yes
	Efavirenz (J05AG03)	42	2.4	34.7	2.6 (1.9-3.5)	Yes	Yes
	Zidovudine (J05AF01)	36	2.2	23.9	2.3 (1.7-3.3)	Yes	Yes
	Ganciclovir (J05AB06)	34	3	44.6	3.3 (2.3-4.7)	Yes	Yes
	Nelfinavir (J05AE04)	26	2.2	17.4	2.4 (1.6-3.6)	Yes	Yes
	Ribavirin (J05AP01)	25	2.7	25.5	2.9 (1.9-4.4)	Yes	Yes
	Ritonavir (J05AE03)	19	2.9	22.1	3.1 (1.9-5.0)	Yes	Yes
	Entecavir (J05AF10)	10	4	20.9	4.7 (2.4-9.3)	No	Yes
	Lamivudine and abacavir (J05AR02)	8	3.1	9.6	3.4 (1.6-7.1)	Yes	Yes
	Valganciclovir (J05AB14)	6	5.2	17.2	6.4 (2.6-15.9)	Yes	Yes
	Simeprevir (J05AP05)	5	9.8	32.5	16.0 (5.1-50.6)	Yes	Yes
	Abacavir/lamivudine/zidovudine sulfate (J05AR04)	4	3.2	4.3	3.6 (1.3-10.3)	Yes	Yes
	Amprenavir (J05AE05)	4	4.7	8.6	5.6 (1.9-16.8)	Yes	Yes
	Saquinavir (J05AE01)	4	3.6	5.4	4.1 (1.4-11.9)	Yes	Yes
	Tebivudine (J05AF11)	3	6.4	9.2	8.4 (2.2-31.8)	No	Yes
J06	Anti-D (rh) immunoglobulin (J06BB01)	5	3.9	8.5	4.5 (1.7-11.7)	No	Yes
L01	Methotrexate (L01BA01)	878	2.4	668.6	2.5 (2.3-2.7)	Yes	Yes
	Vincristine (L01CA02)	649	2.8	728.4	3.0 (2.8-3.3)	Yes	Yes
	Cyclophosphamide (L01AA01)	490	2.3	342	2.4 (2.2-2.6)	Yes	Yes
	Etoposide (L01CB01)	378	2.2	249.6	2.3 (2.1-2.6)	Yes	Yes
	Cytarabine (L01BC01)	344	2.6	349.4	2.8 (2.5-3.2)	Yes	Yes
	Mercaptopurine (L01BB02)	240	3	318	3.2 (2.8-3.7)	Yes	Yes
	Daunorubicin (L01DB02)	207	3.3	332.1	3.6 (3.1-4.2)	Yes	Yes
	Pegaspargase (L01XX24)	180	3.9	404.4	4.5 (3.9-5.3)	No	Yes
	Asparaginase (L01XX02)	163	2.6	165.7	2.8 (2.4-3.3)	No	Yes
	Cisplatin (L01XA01)	155	2.1	95.8	2.3 (1.9-2.7)	No	Yes
	Busulfan (L01AB01)	125	2.5	113.9	2.7 (2.2-3.2)	Yes	Yes

Thiotepa (L01AC01)	85	3.2	129.9	3.5 (2.8-4.4)	Yes	Yes
Actinomycin (L01DA01)	79	2.8	91.7	3.0 (2.4-3.8)	Yes	Yes
Clofarabine (L01BB06)	76	4.1	178.2	4.7 (3.7-6.0)	No	Yes
Imatinib (L01XE01)	75	2.5	65.5	2.6 (2.1-3.3)	Yes	Yes
Tioguanine (L01BB03)	44	3.3	73	3.7 (2.7-5.1)	Yes	Yes
Bortezomib (L01XX32)	37	3.2	55.1	3.5 (2.5-5.0)	Yes	Yes
Vinblastine (L01CA01)	35	2.6	35.3	2.8 (2.0-4.0)	Yes	Yes
Mitoxantrone (L01DB07)	32	2.1	18.2	2.2 (1.5-3.2)	Yes	Yes
Gemcitabine (L01BC05)	26	2.6	24.5	2.8 (1.8-4.2)	Yes	Yes
Fluorouracil (L01BC02)	25	2.3	18.8	2.5 (1.7-3.8)	Yes	Yes
Hydroxycarbamide (L01XX05)	24	2.3	17.1	2.4 (1.6-3.7)	Yes	Yes
Crizotinib (L01XE16)	23	4.6	63.4	5.4 (3.5-8.6)	Yes	Yes
Sorafenib (L01XE05)	23	2.8	26.8	3.1 (2.0-4.8)	Yes	Yes
Paclitaxel (L01CD01)	20	3	25.6	3.3 (2.1-5.2)	Yes	Yes
Nilotinib (L01XE08)	16	3.8	32.1	4.4 (2.6-7.5)	Yes	Yes
Oxaliplatin (L01XA03)	16	3.6	29.4	4.1 (2.4-7.0)	No	Yes
Carmustine (L01AD01)	15	5.6	54.3	7.0 (3.9-12.5)	Yes	Yes
Melphalan (L01AA03)	14	2	6.7	2.1 (1.2-3.7)	No	Yes
Fludarabine (L01BB05)	13	3	16	3.3 (1.8-5.9)	No	Yes
Darafenib (L01XE23)	13	2.9	14.7	3.1 (1.8-5.6)	Yes	Yes
Docetaxel (L01CD02)	12	2.1	6.1	2.2 (1.2-4.0)	Yes	Yes
Lomustine (L01AD02)	11	2.9	12.1	3.1 (1.7-5.9)	Yes	Yes
Pazopanib (L01XE11)	11	2.3	7.5	2.5 (1.3-4.7)	Yes	Yes
Vandetanib (L01XE12)	11	14.3	128.9	35.3 (13.7-91.1)	Yes	Yes
Ruxolitinib (L01XE18)	10	2.7	9.4	2.9 (1.5-5.6)	Yes	Yes
Dasatinib (L01XE06)	9	3.1	11.3	3.4 (1.7-6.9)	Yes	Yes
Cladribine (L01BB04)	9	2.8	9.2	3.1 (1.5-6.1)	No	Yes
Ceritinib (L01XE28)	8	6.7	34.8	9.0 (4.0-20.4)	Yes	Yes
Gemtuzumab (L01XC05)	8	3	9.3	3.3 (1.6-7.0)	No	Yes
Vinorelbine (L01CA04)	8	2.3	5	2.5 (1.2-5.1)	Yes	Yes
Inotuzumab ozogamicin (L01XC26)	7	5	19.3	6.0 (2.6-13.9)	No	Yes
Dinutuximab (L01XC16)	7	2.5	4.9	2.6 (1.2-5.7)	No	Yes
Erlotinib (L01XE03)	5	3.1	5.3	3.4 (1.3-8.7)	Yes	Yes
Ribociclib (L01XE42)	5	6.9	20.5	9.4 (3.3-26.6)	Yes	Yes
Trabectedin (L01CX01)	5	16.8	61.8	56.2 (10.9-289.5)	Yes	Yes

	Sunitinib (L01XE04)	5	5.6	15.2	7.0 (2.6-19.2)	Yes	Yes
	Carboplatin (L01XA02)	4	3.4	4.7	3.7 (1.3-10.8)	No	Yes
	Decitabine (L01BC08)	4	3.5	5	3.9 (1.4-11.3)	Yes	Yes
	Ibrutinib (L01XE27)	4	4.7	8.6	5.6 (1.9-16.8)	Yes	Yes
	Panitumumab (L01XC08)	4	9.4	23.1	15.0 (4.2-53.1)	Yes	Yes
	Gefitinib (L01XE02)	4	5.2	10.2	6.4 (2.1-19.5)	Yes	Yes
	Nelarabine (L01BB07)	3	5.9	8.1	7.5 (2.0-27.7)	Yes	Yes
	Aclarubicin (L01DB04)	3	11.7	20.6	22.5 (4.5-111.3)	Yes	Yes
L03	Interferon alpha-2b (L03AB05)	11	4.8	30.5	5.7 (3.0-11.2)	Yes	Yes
	Peginterferon alfa-2b (L03AB10)	10	2.2	5.9	2.4 (1.2-4.5)	Yes	Yes
	filgrastim (L03AA02)	61	2.5	56.2	2.7 (2.1-3.5)	Yes	Yes
L04	Tacrolimus (L04AD02)	427	2.3	310.2	2.4 (2.2-2.7)	Yes	Yes
	Ciclosporin (L04AD01)	301	2.1	177.9	2.2 (2.0-2.5)	Yes	Yes
	Sirolimus (L04AA10)	74	2.5	67.6	2.7 (2.1-3.4)	Yes	Yes
	Tocilizumab (L04AC07)	71	2.9	91.9	3.2 (2.5-4.1)	No	Yes
	Anakinra (L04AC03)	51	2.4	41.6	2.6 (1.9-3.4)	No	Yes
	Basiliximab (L04AC02)	39	2.9	50.2	3.2 (2.3-4.5)	No	Yes
	Antithymocyte immunoglobulin (rabbit) (L04AA04)	24	2.8	26.6	3.0 (2.0-4.6)	No	Yes
	Leflunomide (L04AA13)	24	2.4	19.4	2.6 (1.7-3.9)	Yes	Yes
	Canakinumab (L04AC08)	18	2.1	9.6	2.2 (1.4-3.6)	No	Yes
	Azathioprine (L04AX01)	18	2.8	20.2	3.1 (1.9-5.0)	Yes	Yes
	Lenalidomide (L04AX04)	4	3.6	5.4	4.1 (1.4-11.9)	No	Yes
	Antilymphocyte immunoglobulin (horse) (L04AA03)	4	3.4	4.7	3.7 (1.3-10.8)	No	Yes
M01	Ketoprofen (M01AE03)	18	2.3	12.2	2.4 (1.5-3.9)	Yes	Yes
	Piroxicam (M01AC01)	14	4.8	40.5	5.8 (3.2-10.5)	Yes	Yes
	Ibuprofen (M01AE01)	13	2.6	11.4	2.8 (1.5-4.9)	Yes	Yes
	Penicillamine (M01CC01)	5	4.2	9.6	4.9 (1.9-12.8)	Yes	Yes
M02	Diclofenac (M02AA15)	12	2.2	6.5	2.3 (1.3-4.1)	Yes	Yes
M04	Allopurinol (M04AA01)	36	3.3	56.6	3.6 (2.6-5.2)	Yes	Yes
	Colchicine (M04AC01)	34	2.9	43.3	3.2 (2.2-4.6)	Yes	Yes
	Febuxostat (M04AA03)	3	5.4	7.1	6.7 (1.9-24.5)	Yes	Yes
M05	Pamidronic acid (M05BA03)	4	3.2	4.3	3.6 (1.3-10.3)	Yes	Yes
M09	Sovaprevir (M09AX09)	14	11.7	132.5	22.5 (10.7-47.2)	Yes	Yes
N02	Paracetamol (N02BE01)	1,365	3.3	1968.2	3.6 (3.4-3.8)	Yes	Yes
	Acetylsalicylic acid (N02BA01)	129	2	66.5	2.1 (1.8-2.5)	Yes	Yes

	Nefopam (N02BG06)	5	10.7	36.2	18.7 (5.7-61.3)	Yes	No
N03	Valproic acid (N03AG01)	511	3	661.3	3.2 (2.9-3.6)	Yes	Yes
	Carbamazepine (N03AF01)	283	2.4	230.4	2.5 (2.2-2.9)	Yes	Yes
	Phenytoin (N03AB02)	179	2.9	229.3	3.2 (2.7-3.7)	Yes	Yes
	Phenobarbital (N03AA02)	147	2.7	164.5	3.0 (2.5-3.5)	Yes	Yes
	Ethosuximide (N03AD01)	34	2.1	19	2.2 (1.5-3.1)	Yes	Yes
	Fosphenytoin (N03AB05)	21	2.7	22.4	3.0 (1.9-4.7)	Yes	Yes
	Felbamate (N03AX10)	16	2.7	15.9	2.9 (1.7-4.9)	Yes	Yes
	Zonisamide (N03AX15)	8	2.8	8.2	3.1 (1.5-6.5)	Yes	Yes
	Valpromide (N03AG02)	7	10.3	51.8	17.5 (6.5-46.9)	Yes	Yes
N04	Trihexyphenidyl hydrochloride (N04AA01)	6	4.7	14.6	5.6 (2.3-13.7)	Yes	Yes
N05	Thiopental sodium (N05CA19)	16	3.1	22	3.5 (2.0-5.9)	Yes	No
	Dexmedetomidine (N05CM18)	10	5.2	31.3	6.4 (3.2-13.0)	Yes	Yes
	Amisulpride (N05AL05)	10	4	20.3	4.6 (2.3-9.1)	Yes	Yes
	Fluphenazine (N05AB02)	10	8.4	60.4	12.5 (5.8-27.1)	Yes	Yes
	Temazepam (N05CD07)	9	2.2	5.3	2.4 (1.2-4.7)	Yes	No
	Levomepromazine (N05AA02)	5	3.7	7.5	4.2 (1.6-10.8)	Yes	Yes
	Perphenazine (N05AB03)	5	3.5	6.7	3.9 (1.5-10.0)	Yes	Yes
	Sulpiride (N05AL01)	5	3.8	8	4.3 (1.7-11.3)	Yes	Yes
N06	Imipramine (N06AA02)	18	2.4	14.5	2.6 (1.6-4.3)	Yes	Yes
	Doxepin (N06AA12)	13	2.7	12.3	2.9 (1.6-5.1)	Yes	Yes
	Moclobemide (N06AG02)	3	8.8	14.3	13.5 (3.2-56.4)	Yes	Yes
	Pemoline (N06BA05)	3	8.8	14.3	13.5 (3.2-56.4)	Yes	Yes
N07	Naltrexone (N07BB04)	10	2.7	9.7	3.0 (1.5-5.7)	Yes	Yes
P01	Pentamidine isethionate (P01CX01)	18	3.6	33.3	4.1 (2.5-6.8)	No	Yes
	Atovaquone (P01AX06)	11	2	5.1	2.1 (1.2-4.0)	No	Yes
	Chloroquine (P01BA01)	10	3.5	15.7	3.9 (2.0-7.6)	Yes	Yes
	Primaquine (P01BA03)	5	9	29.3	14.0 (4.6-42.9)	Yes	No
	Pyrimethamine (P01BD01)	4	3.1	4	3.5 (1.2-9.9)	Yes	No
P02	Albendazole (P02CA03)	20	2.9	24.3	3.2 (2.0-5.1)	Yes	Yes
	Praziquantel (P02BA01)	5	5.1	13.2	6.2 (2.3-16.8)	Yes	Yes
V03	Deferasirox (V03AC03)	212	4.5	592.3	5.3 (4.6-6.2)	Yes	Yes
	Dexrazoxane (V03AF02)	23	4.2	55.6	4.9 (3.1-7.7)	Yes	Yes
	Desferrioxamine (V03AC01)	19	3.9	39.4	4.5 (2.7-7.3)	Yes	Yes
	Deferiprone (V03AC02)	9	3	10.6	3.3 (1.6-6.7)	Yes	Yes

	Rasburicase (V03AF07)	8	3.2	10.7	3.6 (1.7-7.6)	No	Yes
	Calcium levofolinate (V03AF04)	3	14.1	25.6	33.7 (5.6-201.6)	Yes	Yes
D01	Terbinafine hydrochloride (D01AE15)	7	2.7	6.3	3.0 (1.3-6.5)	Yes	Yes
D10	Isotretinoin (D10AD04)	182	2.4	146.5	2.5 (2.2-2.9)	Yes	Yes
G03	norgestimate and ethinylestradiol (G03AA11)	14	2.6	12.6	2.8 (1.6-4.9)	Yes	Yes
R03	Pranlukast (R03DC02)	6	3.3	7.7	3.6 (1.5-8.6)	No	Yes
R05	Mesna (R05CB05)	35	2.5	30.4	2.6 (1.9-3.8)	No	Yes
	Acetylcysteine (R05CB01)	19	2.8	20.3	3.0 (1.9-4.8)	Yes	No
R06	Ketotifen (R06AX17)	10	2.3	6.5	2.5 (1.3-4.7)	Yes	Yes
NA	Sodium valproate	67	3.4	112.7	3.7 (2.9-4.9)	Yes	Yes
	Phenylbutyric acid	11	10.3	87.2	17.7 (8.0-38.9)	Yes	Yes
	Teceleukin	8	20.9	137.9	179.8 (22.5-1,437.4)	No	Yes
	Omega-3-Acid Ethyl Esters	8	5.9	28.8	7.5 (3.4-16.7)	No	Yes
	Aminosalicylic acid	8	3.5	12.7	4.0 (1.9-8.5)	Yes	Yes
	Adenosine deaminase	8	3.6	13.2	4.1 (1.9-8.7)	No	Yes
	Butalbital/paracetamol/caffeine	7	5.7	23.4	7.1 (3.1-16.7)	Yes	Yes
	Loxoprofen	6	3.4	8.4	3.9 (1.6-9.2)	Yes	Yes
	Mizoribine	6	2.6	4.6	2.8 (1.2-6.6)	No	Yes
	Penicillin V potassium	5	5.6	15.2	7.0 (2.6-19.2)	Yes	Yes
	Propoxyphene napsylate	4	15.6	43	44.9 (8.2-245.3)	Yes	Yes
	Teprenone	4	3.9	6.3	4.5 (1.5-13.1)	Yes	Yes
	Medium-chain triglycerides	3	4.1	4.5	4.8 (1.4-16.7)	Yes	Yes

AEs: adverse events, ATC: anatomical therapeutic chemical classification, PRR: proportional reporting ratio, ROR: reporting odds ratio
